# Autonomous Surface and Underwater Vehicles as Effective Ecosystem Monitoring and Research Platforms in the Arctic—The Glider Project †

**DOI:** 10.3390/s21206752

**Published:** 2021-10-12

**Authors:** Lionel Camus, Hector Andrade, Ana Sofia Aniceto, Magnus Aune, Kanchana Bandara, Sünnje Linnéa Basedow, Kai Håkon Christensen, Jeremy Cook, Malin Daase, Katherine Dunlop, Stig Falk-Petersen, Peer Fietzek, Gro Fonnes, Peygham Ghaffari, Geir Gramvik, Inger Graves, Daniel Hayes, Tor Langeland, Harald Lura, Trond Kristiansen Marin, Ole Anders Nøst, David Peddie, Joel Pederick, Geir Pedersen, Ann Kristin Sperrevik, Kai Sørensen, Luca Tassara, Sigurd Tjøstheim, Vigdis Tverberg, Salve Dahle

**Affiliations:** 1 Akvaplan-niva AS, 9007 Tromsø, Norway; maa@akvaplan.niva.no (M.A.); sfp@akvaplan.niva.no (S.F.-P.); pgh@akvaplan.niva.no (P.G.); oan@akvaplan.niva.no (O.A.N.); luca.tassara@akvaplan.niva.no (L.T.); sd@akvaplan.niva.no (S.D.); 2 Institute of Marine Research, 9007 Tromsø, Norway; hector.andrade@hi.no (H.A.); katherine.mary.dunlop@hi.no (K.D.); 3 The Norwegian College of Fishery Science, Faculty of Fisheries and Bioeconomics, UiT—The Arctic University of Norway, 9037 Tromsø, Norway; ana.s.aniceto@uit.no; 4 Faculty for Bioscience and Aquaculture, Nord University, 8026 Bodø, Norway; kanchana.bandara@nord.no (K.B.); vigdis.tverberg@nord.no (V.T.); 5 Department of Arctic and Marine Biology, Faculty of Biosciences, Fisheries and Economics, UiT The Arctic University of Norway, 9037 Tromsø, Norway; sunnje.basedow@uit.no (S.L.B.); malin.daase@uit.no (M.D.); 6 R&D Department, Norwegian Meteorological Institute, 0371 Oslo, Norway; kaihc@met.no (K.H.C.); annks@met.no (A.K.S.); 7 NORCE Norwegian Research Center, 5008 Bergen, Norway; jeco@norceresearch.no (J.C.); grfo@norceresearch.no (G.F.); tola@norceresearch.no (T.L.); gepe@norceresearch.no (G.P.); 8 Kongsberg Maritime Germany GmbH, 22529 Hamburg, Germany; peer.fietzek@km.kongsberg.com; 9 Kongsberg Digital, 3616 Kongsberg, Norway; Geir.gramvik@kdi.kongsberg.com (G.G.); sigurd.tjostheim@kdi.kongsberg.com (S.T.); 10 Aanderaa Xylem, 5225 Nesttun, Norway; Inger.Graves@Xyleminc.com; 11 Cyprus Sub Sea Consulting & Services, 2326 Nicosia, Cyprus; hayesdan@cyprus-subsea.com; 12 ConocoPhillips Skandinavia AS, 4056 Tananger, Norway; Harald.Lura@conocophillips.com; 13 Marin Biogeochemistry and Oceanography, NIVA, 0579 Oslo, Norway; trond.kristiansen@niva.no (T.K.M.); kai.sorensen@niva.no (K.S.); 14 Offshore Sensing AS, 5072 Bergen, Norway; david@sailbuoy.no; 15 Maritime Robotics AS, 7010 Trondheim, Norway; joel@maritimerobotics.com

**Keywords:** glider, remote sensing, ecosystem monitoring, Lofoten–Vesterålen

## Abstract

Effective ocean management requires integrated and sustainable ocean observing systems enabling us to map and understand ecosystem properties and the effects of human activities. Autonomous subsurface and surface vehicles, here collectively referred to as “gliders”, are part of such ocean observing systems providing high spatiotemporal resolution. In this paper, we present some of the results achieved through the project “Unmanned ocean vehicles, a flexible and cost-efficient offshore monitoring and data management approach—GLIDER”. In this project, three autonomous surface and underwater vehicles were deployed along the Lofoten–Vesterålen (LoVe) shelf-slope-oceanic system, in Arctic Norway. The aim of this effort was to test whether gliders equipped with novel sensors could effectively perform ecosystem surveys by recording physical, biogeochemical, and biological data simultaneously. From March to September 2018, a period of high biological activity in the area, the gliders were able to record a set of environmental parameters, including temperature, salinity, and oxygen, map the spatiotemporal distribution of zooplankton, and record cetacean vocalizations and anthropogenic noise. A subset of these parameters was effectively employed in near-real-time data assimilative ocean circulation models, improving their local predictive skills. The results presented here demonstrate that autonomous gliders can be effective long-term, remote, noninvasive ecosystem monitoring and research platforms capable of operating in high-latitude marine ecosystems. Accordingly, these platforms can record high-quality baseline environmental data in areas where extractive activities are planned and provide much-needed information for operational and management purposes.

## 1. Introduction

Oceans cover up to 71% of the earth’s surface, regulate the global climate system, supply more than half the biosphere’s oxygen, and sustain the livelihood of billions of people worldwide [1,2,3,4,5,6]. The ongoing decline in land-based resources is causing increased demand for marine resources such as food and energy and is expanding human marine activities including transportation and recreation, which ultimately threaten the ocean’s health [6,7,8,9]. Effective ocean management requires integrated and sustainable ocean observing systems, which enable us to better understand ocean processes and the impacts of human activities [6,10]. Autonomous subsurface and surface vehicles, here collectively referred to as |“gliders”, are advanced ocean observing systems providing high spatiotemporal resolution [10,11,12,13,14,15]. Equipped with energy-efficient sensors, innovative communication means, and data management systems, these vehicles are commanded remotely, and report results to land in near-real-time, providing a new set of tools to efficiently collect ecological and commercially relevant data from the open ocean and coastal areas. Through the project “Unmanned ocean vehicles, a flexible and cost-efficient offshore monitoring and data management approach”, three different gliders were deployed from March to September 2018, along the Lofoten–Vesterålen (LoVe) shelf-slope-oceanic system in Arctic Norway. This period covered the annual blooms of primary and secondary production, which in turn sustain high fish density aggregations in spawning grounds where cetaceans also feed [11,16,17,18,19]. The highly productive LoVe area is of prime interest to develop the national blue economy, with stakeholders including fisheries, offshore energy, aquaculture, shipping, and tourism. As such, the area is prone to increased anthropogenic pressure and concurrent environmental changes [20]. Current and future ocean-based value creation depends on sustainable use of the ocean’s resources for which environmental monitoring and research are fundamental [20]. There is a need to gain knowledge about the LoVe ecosystem to develop appropriate management approaches to ensure environmental quality, business sustainability [12,21], and good governance. Building this knowledge requires additional and persistent data collection that cannot rely solely on sampling by traditional vessels due to practical and economic reasons. Currently, autonomous continuous long-term in situ monitoring programs exist for the LoVe region thanks to an underwater observatory system that employs crawler and stationary platforms to study ecological processes in the deep sea (https://love.equinor.com/) (accessed on 7 October 2021) [12,22,23,24,25]. The deep sea, however, is a highly dynamic environment where benthic ecosystems are interconnected with the water column and the surface through the exchange of energy, mass, or nutrients [26,27,28]. Thus, knowledge of a wide variety of ecological and biological variables is needed to improve ecosystem monitoring, from the seabed to the surface. To achieve such monitoring capabilities of the entire water column across a wide spatiotemporal spectrum, a spatial hierarchy of clusters and networks should integrate various observation technologies [29,30]. 

The goal of the GLIDER project was to test three gliders equipped with sensors capable of collecting physical, biogeochemical, and biological data simultaneously, performing an integrated ecosystem survey across different depths and a wide geographical area. At the same time, data collected by the gliders were incorporated in near-real-time into a data assimilative ocean circulation model to test whether the model forecast could be improved for the LoVe region, where geomorphological challenges are brought about by a complex coastline, the presence of fjords, and strong seasonal freshwater input. A data management e-platform was developed to ensure data storage and visualization for researchers and managers. 

In this paper, we present the project concept and review some of the initial results achieved after a six-month deployment along the LoVe shelf-slope. These findings, initially presented at the OCEAN 2019 conference (Marseille, 17–20 June) [21] are expanded here and demonstrate the cost-efficient, long-term, low invasive, and autonomous monitoring and research capabilities of gliders in collecting high-quality environmental and biological data simultaneously. Together, gliders and other autonomous robotic seafloor infrastructure offer unique ecosystem monitoring capabilities for the LoVe region. 

## 2. Materials and Methods

### 2.1. Surface and Subsurface Vehicles

**Figure 1:**
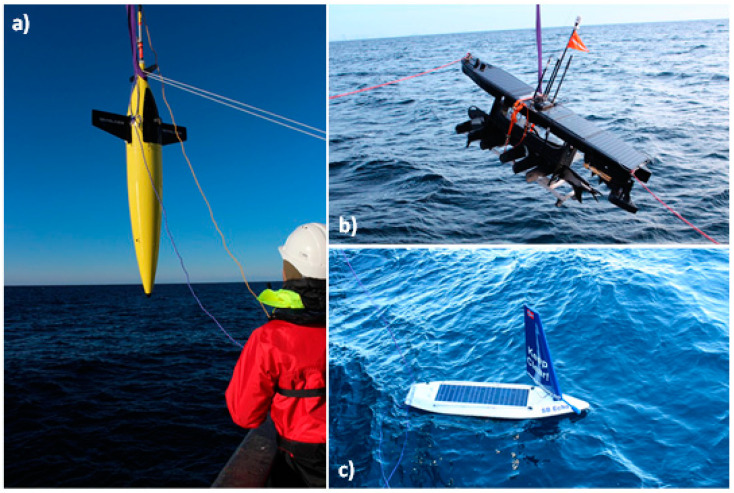
Surface and subsurface platforms employed in the GLIDER project: (**a**) Seaglider^®^ (Huntington Ingalls Industries; formerly Kongsberg Maritime); (**b**) Wave Glider SV3 (Liquid Robotics); and (**c**) Sailbuoy (Offshore Sensing).

The GLIDER project deployed three different surface and subsurface platforms: a Seaglider^®^ M1 (Huntington Ingalls Industries Inc.; formerly Kongsberg Maritime), a Wave Glider SV3 (Liquid Robotics), and a Sailbuoy (SB, Offshore Sensing) (Figure 1). The Seaglider^®^ is an autonomous underwater vehicle that uses changes in buoyancy to move through the water column in a sawtooth pattern while collecting data from the ocean surface to a programmed depth and back [31]. The vehicle is powered by Lithium Sulfuryl chloride primary batteries 17 MJ allowing operations in the range of several thousand kilometers and several months depending on configuration and payloads. The operational depth range is between 50 and 1000 m. The vehicle is 1.8–2 m long depending on the configuration and has a maximal diameter of 0.3 m. The wingspan is 1 m, and the antenna mast length varies between 0.43 and 1 m. The typical operation speed is 0.5 knots. A 2-way Iridium communication system was used for navigation and transmitting data (for further details [32]).

The Wave Glider SV3 is a long-duration unmanned surface vehicle utilizing wave energy for propulsion [33]. The Wave Glider consists of a surface float tethered with an 8 m umbilical cable to a subsurface glider. The surface float houses the command-and-control unit for communication, navigation, and power systems, containing a 980 Wh lithium-ion battery pack charged by solar panels (150 W). The maximum payload weight is 59 kg, and the maximum and average water speed are 3 and 1.8 knots, respectively. Additionally, the Wave Glider has an electric propulsion system to increase mobility in challenging ocean conditions. For the current project, a 2-way Iridium communication system for data transmission and navigational instructions was used (for further details [34]). 

The Sailbuoy (Offshore Sensing) is a long-duration unmanned surface vehicle using wind for propulsion [35]. The vehicle employed in the GLIDER project was 2 m × 0.5 m × 1.3 m (length, beam, height), with a displacement of 60 kg and payload up to 10 kg. This vehicle may operate for several months with average speeds of 1–3 knots. Navigable wind speeds are 3–30 m/s (tested up to 30 m/s) and the navigable wave height is up to about 15 m. Autopilot and sensor payloads are powered by separate battery packs charged by solar panels (30 W). Two-way Iridium communication was used for navigational instructions and for transmitting data (for further details [36]). In the GLIDER project, the three vehicles were equipped with current state-of-the-art sensors to measure biogeochemical and biological parameters (Table 1). 

### 2.2. GLIDER Deployment and Data Collection

The three vehicles were deployed along LoVe from March to September 2018, a period of high biological activity (see below). The vehicles were piloted by Maritime Robotics (Wave Glider), Offshore Sensing (Sailbuoy), and Cyprus SubSea Consulting and Services (Seaglider^®^). Initial deployment of the vehicles occurred from the R/V Håkon Mosby, offshore Bodø, Norway. The vehicles were retrieved twice during the survey period to repair a technical problem with the memory storage, and at the same time, offload the acoustic data. A well-known challenge with autonomous acoustic surveys is the identification of the acoustic targets based only their acoustic signal. To identify targets, additional sampling might be performed by trawl, net, or occasionally optical sampling [15,37,38]. Zooplankton composition, abundance, and vertical distribution in an area overlapping with the track of the Sailbuoy in June 2018 (6 stations west of Vesterålen, 10 stations north on Tromsøflaket) was assessed during an independent research cruise aboard RV Helmer Hanssen (see https://lofoten-research.no/ accessed on 7 October 2021) using a Hydrobios Multinet (mesh size 180 µm, opening area 0.25 m^2^), a Tucker Trawl (1500 µm, 1 m^2^), a Video Plankton Recorder (Seascan Inc.), and a Laser Optical Plankton Counter (ODIM-Brooke Ocean Rolls Royce Canada Ltd.) following the methods described in [39]. 

#### 2.2.1. Study Site

**Figure 2:**
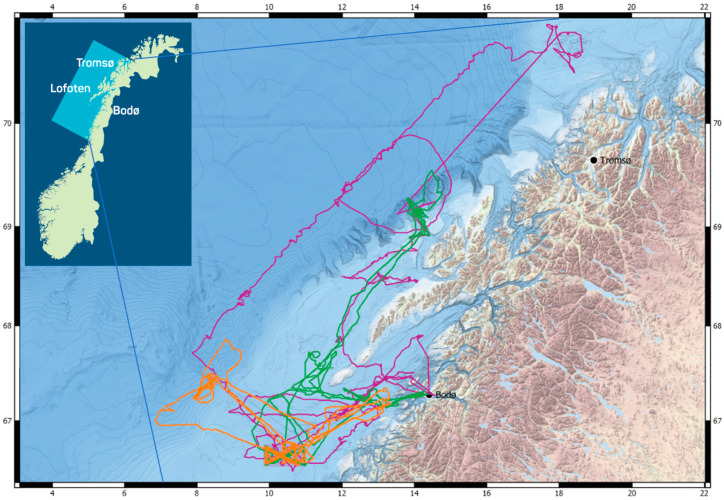
Extended Lofoten–Vesterålen (LoVe) region (insert) and tracks of surface and subsurface platforms deployed in the GLIDER project, from March to September 2018 (Orange: Seaglider^®^, Green: Wave Glider, Purple: Sailbuoy).

The study site was the shelf-slope-oceanic ecosystem of LoVe (Figure 2). The area is considered rich in biological resources and was characterized as “particularly vulnerable” by the Norwegian authorities [20]. In this area, the phytoplankton spring bloom starts in the fjords by the end of March, progresses to the shelf by April–May, and extends to the basin by May–June. The increased primary production enhances biological activity from the seabed and across the entire water column. The increase in chlorophyll concentration prompts polyp activity in corals [23] and is followed by the ascension to the surface of the overwintering copepod *Calanus finmarchicus*, the most abundant zooplankton in the region. *C. finmarchicus* feeds on the phytoplankton production and reproduces during spring and summer [19,39,40]. The marine zooplankton community provides important trophic linking between the microalgae community and higher trophic level species, sustaining major fish stocks, including the highly abundant stocks of cod *Gadus morhua* (i.e., the Northeast Arctic cod), herring *Clupea harengus* (i.e., the Norwegian spring spawning herring), and blue whiting *Micromesistius poutassou* [18,41,42,43]. The LoVe region also provides important spawning grounds and larval areas for the commercial fish stocks of the Barents Sea and adjacent waters, including the Northeast Arctic haddock *Melanogrammus aeglefinus* stock, the Norwegian spring-spawning herring *Clupea harengus* stock, and the Northeast Arctic cod *Gadus morhua* stock [43]. Cetaceans were also reported in the area [11,44], with both humpback whale *Megaptera novaeangliae* and killer whale *Orcinus orca* feeding on herring aggregations [44]. The Lofoten Basin is the most eddy-rich region in the Norwegian Sea [45]. Mesoscale eddies transport phytoplankton and key ecosystem species, such as *C. finmarchicus* and mesopelagic fish, across the shelf. Near the shore, tidal currents play a role in transporting cod eggs northward past the Lofoten Peninsula [46], while the Norwegian Coastal Current (NCC) and the Norwegian Atlantic Current (NwAC) play the main role in transporting eggs into the Barents Sea [19,47]. For example, cod spawned in the LoVe region was found later residing in the Svalbard fjords [48].

#### 2.2.2. Echo Sounder Mapping of Shallow Zooplankton Layers

The Wave Glider and the Sailbuoy were equipped with the Simrad WBT Mini (Kongsberg Maritime), a compact and energy-efficient scientific echo sounder in the EK80 family. The Wide Band Transceiver (WBT) Mini can work with continuous wave (CW) and broadband frequency modulated (FM) pulses. Although initially designed for autonomous operations on stationary platforms, it has found widespread use on small and large autonomous mobile vehicles. The Wave Glider was fitted with two split-beam transducers, Simrad ES70-18CD and ES333-7CDK with a center frequency of 70 and 333 kHz, respectively. The Sailbuoy was fitted with one split-beam transducer, the Simrad ES333-7CDK. The center frequencies were chosen to maximize the signal strength from copepods (*C. finmarchicus* in particular) and to continuously monitor the shallow layers of copepods throughout the duration of the mission. In this mission, control of the echo sounder data collection was available on both vehicles through the vehicles’ two-way Iridium satellite communication (for additional details of the echo sounder integration, operation, and data collected, see [49]).

#### 2.2.3. Sea Mammal Vocalizations and Anthropogenic Noise

To passively scan for marine mammal vocalizations and anthropogenic sound, two of the three automated vehicles were used. The Seaglider^®^ was equipped with an Observer/AMAR G4 from JASCO [11,50,51] and recorded continuously down to 100 m depth. The Wave Glider was equipped with a hydrophone SoundTrap from OceanInstruments. To date, only the Seaglider^®^ data were analyzed. To determine the different types of biological and anthropogenic sounds that occur in the LoVe area, the recordings were inspected visually and aurally using automatic detection and visualization software (i.e., PAMguard [52] and Raven [53]). Ship noise consists of broadband noise depending on the size and speed of the vessels, but most of the energy is at low frequencies (<1 kHz). Typically, the sound of a ship passing by can last for tens of minutes. Automatic detectors and manual spectrogram analysis identified biological sounds as well as weather events, vessel passages, and seismic activities. Ambient noise was defined as all sound recorded, except sound associated with a specified signal and the self-noise produced by the vehicle (e.g., pitch/roll maneuvers, apogee pump activity, etc.). Prior to data analysis, all self-noise was detected and removed from the dataset using template matching [11]. 

#### 2.2.4. Ocean Modelling

The continental slope off Norway is a transport passage for the warm-saline Norwegian Atlantic Current (NwAC) towards the Arctic Ocean. Here, the current is primarily guided by the continental slope. Hence, mesoscale or sub-mesoscale eddies are considered the primary mechanism in lateral heat transport. Indeed, observations and models indicate strong flow field variability in this region [45,54]. The complex flow field and geometry of coastal and fjord systems entails not only a high spatial resolution of the ocean circulation model but also associated high-resolution observations and forcing fields, e.g., from numerical weather prediction models. Data assimilation (DA) using near-real-time observations is much needed to improve predictive skills in such models. Accordingly, to better understand the physics of the ocean along the coastal zone of the LoVe region, meteorological and oceanographic data collected by the three gliders were assimilated into a regional version of the ROMS model (NorShelf-2.4km). The NorShelf-2.4km model already assimilates data from diverse sources, e.g., sea surface temperature, high-frequency radars, hydrography provided by CTD sections from occasional research cruises, FerryBox data, and ARGO floats. This model runs operationally at the Norwegian Meteorological Institute (MET Norway) using four-dimensional variational data assimilation (4D-Var [55]). ROMS provides several formulations of 4D-Var; NorShelf-2.4km uses the physical space analysis system (PSAS). Within this setup, the assimilation window was two days, which was chosen based on previous 4D-Var experiments on the coast of Norway with a 2.4km resolution [56]. To benefit from the improved hydrography and circulation that the DA in NorShelf-2.4km provides, a very high-resolution unstructured model was nested as a grid into the NorShelf-2.4km [57]. This allowed for the introduction of a large number of rivers (more than 7000 large and small rivers) and positioned them correctly on the coastline. Here, we adopted a 3D version of the Finite-Volume Community Ocean Model (FVCOM [58]) and imposed a variable spatial resolution from 30 m along the coast and straits to 2.4 km at the boundaries. Hence, by imposing high-quality boundary conditions, an enhanced picture of the ambient hydrodynamical processes was achieved. In order to assess the skill of NorShelf-2.4km compared with MET Norway’s high-resolution NorKyst-800m forecast model, which does not employ data assimilation, we compared T-S diagrams. 

### 2.3. GLIDER Data Management Platform

**Figure 3:**
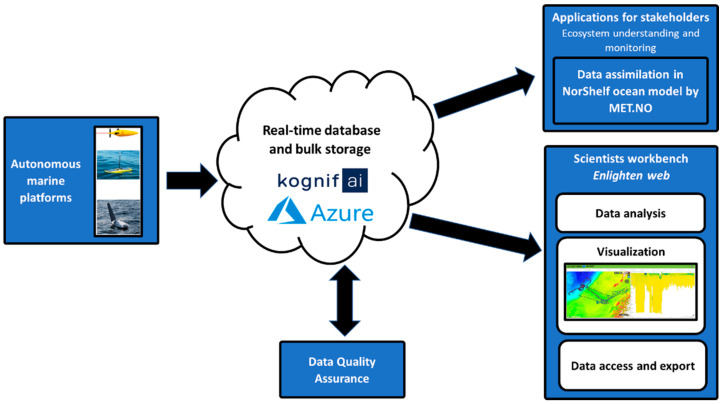
Overview of the e-infrastructure implemented in the GLIDER project. The gliders send time-series data via satellite links, which are stored in the Kognifai digital ecosystem. Here, servers at Niva automatically perform data quality control and the data are available through a web portal. The NorShelf ocean model also assimilates data, improving ocean predictions. Satellite links are a potential communication resource not used in their presented work due to high costs.

To manage the data collected by the gliders, a data management platform “GLIDER” was developed by the Norwegian Research Centre (NORCE), Kongsberg Digital (KDI), and the Norwegian Institute for Water Research (NIVA). The platform was designed to perform quality control through a range of tests, ensuring high-quality data access to researchers and end-users. The data management platform provides continuous tracking of autonomous measurement platforms during surveys with real-time access to time series of measured data (e.g., temperature, salinity, conductivity, and fluorescence). The gliders send time-series data via satellite links (Figure 3). The solution for storing the time series is based on the Kognifai digital ecosystem provided by KDI. The solution consists of an asset database in addition to the time-series database. The metadata for the gliders, instruments, and sensors are stored in the asset database [59].

**Figure 4:**
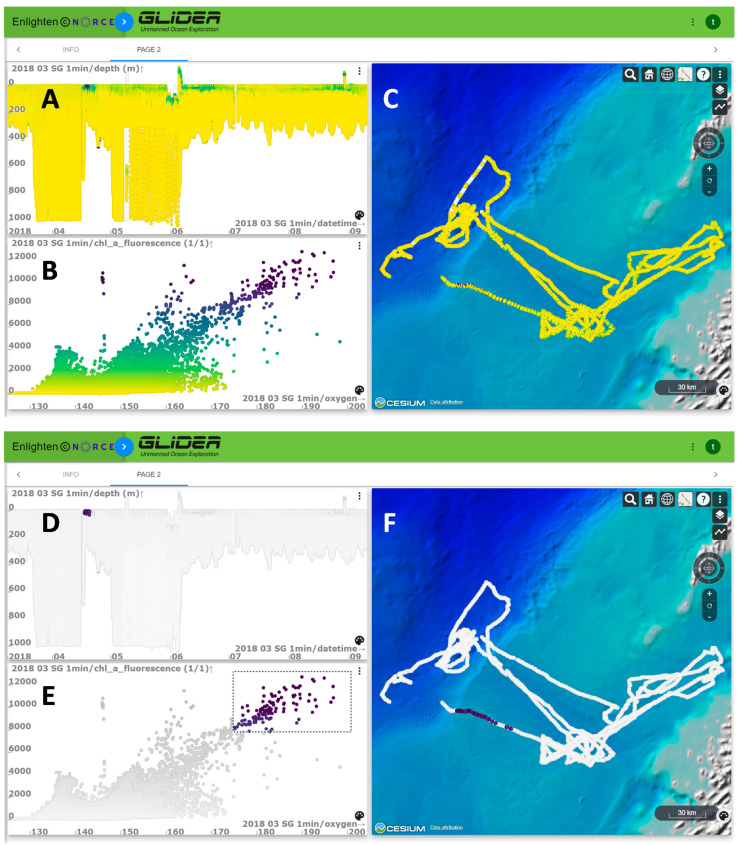
Example plots from the GLIDER web portal showing down-sampled data collected by the Seaglider^®^ in the 2018 survey. In all plots, color on data points corresponds to the level of chlorophyll-a (CHl-a, fluorescence). (**A**) Depth along the Seaglider^®^ path (x-axis is time, y-axis is depth). (**B**) Correlation between oxygen (x-axis) and CHl-a (y-axis). (**C**) Path travelled by Seaglider^®^ for which data are displayed. Bottom panel shows the depth (**D**) and CHl-a-oxygen correlation (**E**) along the Seaglider^®^ path (**F**) after the user applied a filter highlighting high CHl-a and oxygen values.

The GLIDER web portal implements interactive visual analysis and covisualization of large multidimensional datasets for exploration of the collected data, giving scientists access to the data stored in these databases. The portal was implemented by adapting Enlighten-web, a web application developed by NORCE, and supports interactive visual analysis and covisualization of large multidimensional datasets. Predefined plots were prepared to follow the data acquisition in real-time and to see a quick overview of the data. Users can easily create and store their own plots for further analysis. Brushing and linking [60] functionality is provided for exploring complex datasets, to discover correlations and interesting properties hidden in the data (Figure 4). Enlighten-web offers interactive performance in brushing and linking on large datasets by utilizing the graphic processing unit on the user’s computer. Several filters can then be combined using Boolean “AND” operators. 

The GLIDER web portal also supports Web Map Services (WMS). This allows the web portal users to contextualize observations by adding map layers from external services, e.g., representing meteorological and oceanographic data.

During a survey, NIVA servers monitor the Kognifai database time series and automatically perform quality control (QC) services. The QC services collect the new data added to the database and perform a set of regionally dependent QC tests. A flag indicating the quality of each data point is written back to the database. The QC tests and algorithms follow the International Copernicus.eu standard [61]. The web portal provides an option to filter and view only good-quality data flagged during the QC process. 

Due to bandwidth and cost limitations, echo sounder and hydrophone data were not transmitted to the time series database via satellite link but stored on-board the gliders. These data files were retrieved at the end of the survey and uploaded to Microsoft Azure Blob Storage. Through the brushing and linking functionality, the GLIDER portal supports selecting relevant bulk data for download, which can be readily incorporated into models. For example, MET Norway uses hydrographic data provided by the gliders for near-real-time assimilation in their ocean model [59].

## 3. Results

### 3.1. GLIDER Environmental Data Recordings

**Figure 5:**
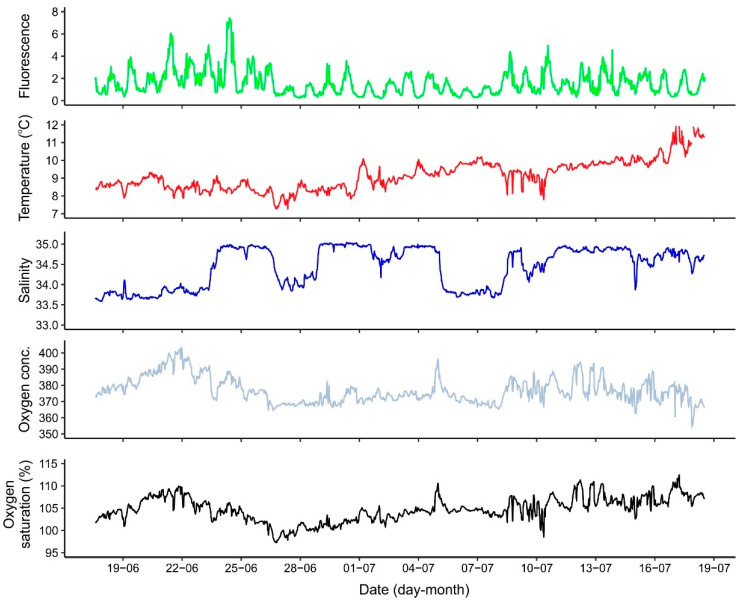
Subset of environmental variables (fluorescence, temperature, salinity, and oxygen) recorded by the Wave Glider during a 1-month period (June–July 2018) across a transect in the LoVe region.

The three gliders effectively recorded data across planned transects in the LoVe region. All data were uploaded into the GLIDER data management platform where scientists could access and download the data for further analysis. For instance, a subset of environmental data recorded by the Wave Glider showed temporal variation and trends in surface fluorescence, temperature, salinity and oxygen during the summer (June–July) (Figure 5). These data were further employed to explore relations between environmental parameters and the spatiotemporal distribution of zooplankton and cetaceans, as well as to improve regional ocean models (see below). 

### 3.2. Echo Sounder Mapping of Shallow Zooplankton Layers

**Figure 6:**
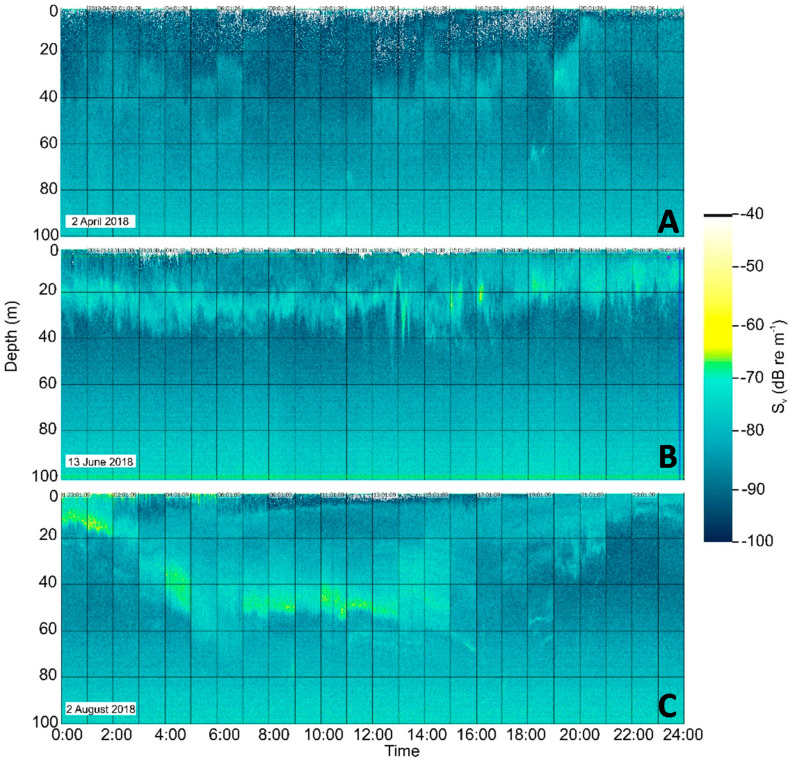
Three examples of echograms of volume backscattering strength (S_V_) at 333 kHz collected by WBT Mini on the Sailbuoy in the upper 100 m and integrated over a 24 h period. The echograms display differences in zooplankton aggregation during the three periods and regions of the 2018 deployment. (**A**) April, deep and thin registrations in Vestfjord, (**B**) June, persistent dense layer in the upper 10–30 m, along the western coast of Vesterålen, (**C**) August, wide vertical distribution with indications of diel vertical migration, along the shelf break.

The Wave Glider and the Sailbuoy successfully collected good quality echo-sounder data along the Norwegian coast from 66.5 N to 70.9 N, from March to September 2018. The data allowed for mapping of the spatiotemporal and vertical distribution and density of zooplankton down to approximately 100 m depth. Using the WBT Mini echo sounder on the Sailbuoy, dense spring zooplankton concentrations were detected throughout the deployment period (Figure 6).

In early spring (April), when copepods ascend from overwintering depths, weak layers of zooplankton were registered from approximately 20–30 m depth. Later in the season (June), a persistent high-density layer of zooplankton was observed in the upper 10–40 m. In late summer (July–August), the zooplankton layers were distributed across a wider depth range, exhibiting diel vertical migration patterns [49]. Stronger individual scatterers were also observed within, below and above the zooplankton layer, likely fish and fish larvae grazing on the zooplankton layer. Net sampling in April and June in adjacent areas revealed that zooplankton composition comprised mainly the copepod *Calanus finmarchicus*, a dominant zooplankton species in the area [39].

The Sailbuoy concurrently measured environmental variables, including temperature, salinity, and oxygen saturation. Surface water temperature and salinity increased from April to July/August while the highest oxygen saturation was found in the period of maximum zooplankton surface layer density (Table 2). The fluorescence measurements recorded by the Wave Glider at 0.5 m revealed a diurnal cycle of the fluorescence throughout the study period, with minimum values recorded at noon, and maximum values at night (Figure 5). This was observed in other studies using gliders (e.g., [62,63,64]) and reflects physiological quenching, an adaptation of photosynthetic organisms to avoid photo damage when exposed to excess light that leads to a massive decrease in in situ fluorescence [65]. 

### 3.3. Sea Mammal Vocalization and Anthropogenic Noise

The Observer/AMARG4 hydrophone installed on the Seaglider^®^ was able to record cetacean vocalizations, anthropogenic operations, and weather events across the LoVe continental shelf break and results from the spring records (March–April 2018) were recently published [11]. In short, delphinids, fin whales *Balaenoptera physalus*, humpback whales *Megaptera novaeangliae,* and sperm whales *Physeter macrocephalus* were identified during the deployment. During the first weeks (March), humpback whale calls were dominant on both the shelf and shelf edge, coinciding with the species’ well-known migration patterns toward breeding grounds [66,67]. From mid-April, during the spring phytoplankton bloom, sperm whales and delphinids were mostly recorded. Sperm whales were mostly detected in more open waters and within a 400 m underwater canyon, coinciding with their preferred foraging habitat [11]. This analysis showed seasonal and spatial variability in marine mammal detections.

**Figure 7:**
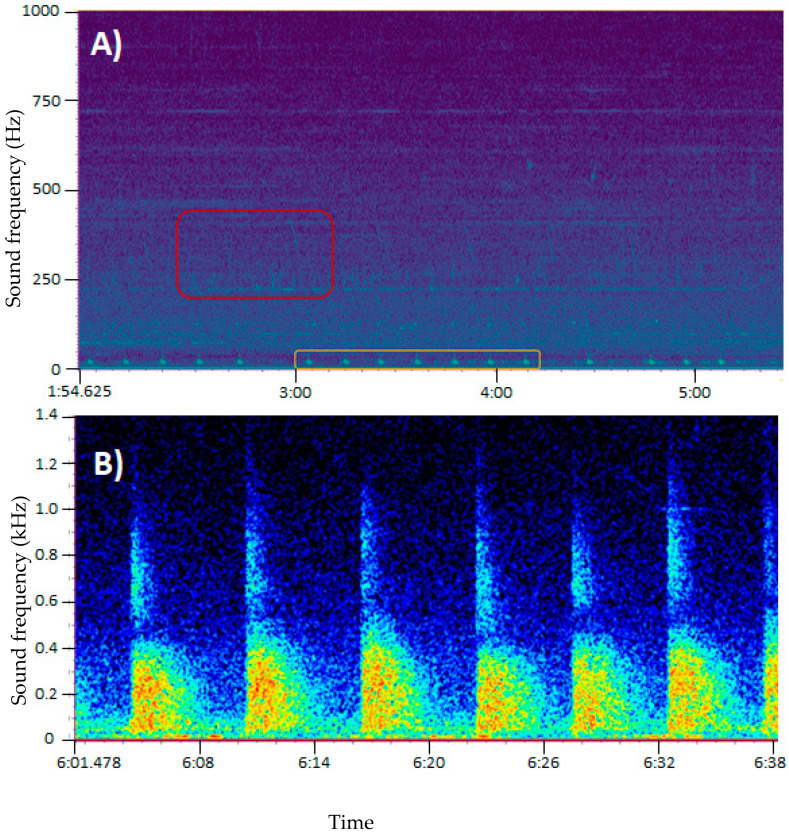
Spectrogram showing the narrow frequency of a humpback whale (red) and a fin whale (orange) vocalization (**A**) and airgun shots from a seismic survey (**B**). Color represents sound from low (dark blue) to high intensity (yellow–red). Data collected with the Seaglider^®^ equipped with an Observer/AMAR G4 from JASCO.

Manual and automatic detection of anthropogenic noise identified sounds that could be attributed to distinct human activities in the area. The most common anthropogenic noises detected were vessel propellers (maritime traffic), seismic surveys, and offshore operations. A preliminary analysis of the entire survey seems to confirm that ship traffic is the most encountered type of anthropogenic sound. However, while ship traffic represents the most frequently recorded source of anthropogenic disturbance, seismic shooting activities were also detected, with some activities overlapping in both time and frequency with marine mammal vocalizations (Figure 7).

### 3.4. Ocean Modeling

**Figure 8:**
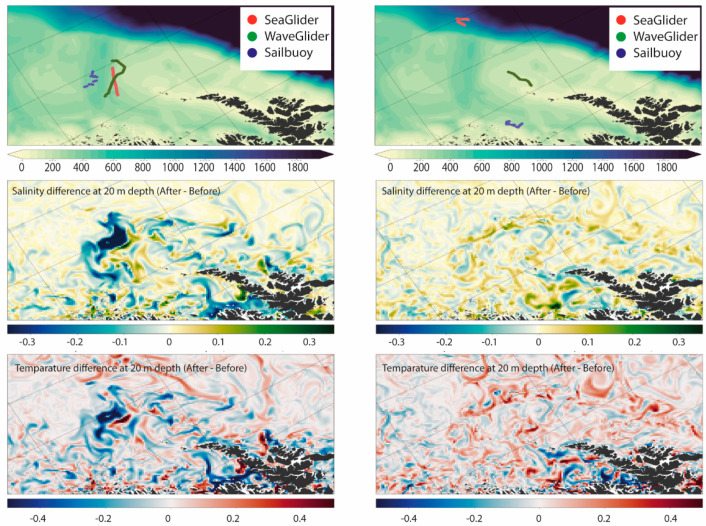
Snapshots (left column: 20 March 2018, right column: 3 April 2018) of glider data assimilation influence on the NorShelf-2.4km model salinity and temperature fields. Upper panel: topography and gliders’ positions, middle panel: salinity difference at 20 m depth, lower panel: temperature difference at 20 m depth [57].

Seaglider^®^, Sailbuoy, and Wave Glider observations were assimilated into the NorShelf-2.4km model daily by ingesting data from the past two days to produce improved initial conditions for the ocean forecast and the nested high-resolution ocean model of the LoVe region. The assimilation of glider observations had a significant impact on the model fields. While the maximum analysis increments occurred near the glider’s locations, glider data assimilation also had a considerable effect on adjusting the remote hydrographical field (Figure 8).

**Figure 9:**
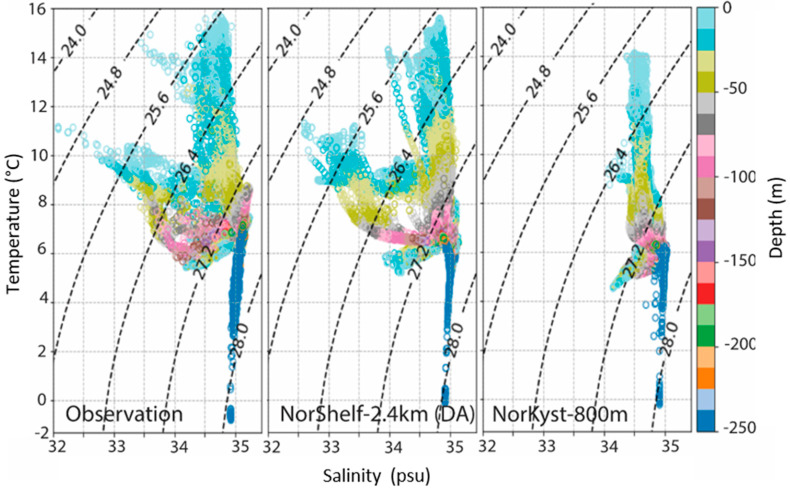
Temperature–salinity diagrams (T-S) comparing observations collected by the gliders (**left panel**), the NorShelf-2.4km model (DA, **central panel**), and the NorKyst-800m model (**right panel**).

The difference between the two models was mainly dominated by salinity (Figure 9). NorShelf-2.4km had a more realistic description of the low-saline water masses associated with the Norwegian Coastal Current and better overall agreement with the observations. Our results indicated that even with relatively limited incorporation of glider observations, the model performance was improved over a larger geographical extent. This functionality of Gliders, in turn, provided a significant gain in adjusting the simulations and may help to constrain small-scale circulation features, such as mesoscale eddies. Gliders can thus be an important complement to the existing observations [57]. The previous ocean model simulation for the LoVe region (METreport-04/2018 ISSN 2387-4201 Oceanography) showed a strong negative bias both in salinity and temperature fields. This could be an indication of an inaccurate freshwater in-flow budget. In the LoVe region, the NwAC meets a narrow continental shelf, where large-scale flow dynamics directly affect the oceanic micro-processes. The multiscale high-resolution setup was helpful in better resolving those processes (for more details, see [57]). 

## 4. Discussion

In this paper, we presented results from the GLIDER project in which two surface and one subsurface vehicle were deployed to collect ecological and environmental data throughout the LoVe region, from March to September 2018. In these months, the gliders recorded data during a period of increased biological activity starting prior to the onset of the spring phytoplankton bloom, zooplankton overwintering ascendance and reproduction, and cetacean migrations. Previously, four operational factors were identified as limiting the use of ocean observatories in ecological and fisheries applications: “(1) limited spatial coverage, (2) limited integration of multiple types of technologies, (3) limitations in the experimental design for in situ studies, and (4) potential unpredicted bias in monitoring outcomes due to the infrastructure’s presence and functioning footprint” [29]. The results presented here demonstrate that gliders can be effectively used as noninvasive ocean observatories for ecosystem monitoring and as research platforms expanding spatial coverage over a wide spatiotemporal scale while integrating multiple types of sensors able to record environmental and biological variables. Gliders thus add to the toolkit of long-term remote noninvasive devices tested in the Norwegian Arctic (e.g., [12,14,68]). Traditionally, autonomous vehicles have mainly been used for collecting physical and chemical data from the oceans. Direct observations of multiple trophic levels have been lacking partly due to the size and power consumption of older scientific echo sounders hampering the deployment onboard autonomous vehicles. The small echo sounders deployed in this project, however, allowed for data collection of zooplankton densities and distribution over the whole deployment period, ensuring detection of the organisms in an undisturbed state. The Sailbuoy survey reliably recorded data over a large spatiotemporal scale and thus provided new insights into plankton distribution patterns and behavior that would not have been possible to observe using traditional ship-based instrumentation alone due to among other issues, the duration and costs of research cruises collecting data daily, over a period of months. Furthermore, while hull-mounted echo sounders generally fail to provide data from the upper 5–20 m of the water column due to strong acoustic reflections from the surface and the ship’s draft that obscures the near-surface region [69], the echo sounder on the Sailbuoy recorded zooplankton densities at shallow depths (2–10 m depth) with little to no negative effects due to mechanical noise [49]. This is because transducers were mounted at a deeper water depth on a surface vessel as compared to gliders. Nevertheless, the quality of the acoustic records in the upper layers did vary with weather conditions as bubble entrainment during periods of strong wind and high wave action disturbed the signal mainly in the upper four meters, but on occasions even down to 25 m. In addition, the acoustic data provided by the Sailbuoy were recorded without introducing light pollution. Recently, it was shown that biological surveys performed in the dark by illuminated ships, may introduce biases on biological sampling, bioacoustic surveys, and possibly stock assessments of commercial and non-commercial species, due to among others, disruption of species diel vertical migration and strong light-escape responses [70,71,72]. This becomes especially problematic in the Arctic polar night, characterized by low irradiance levels day and night. 

The glider echo sounder survey aimed at mapping zooplankton densities in the uppermost 50 m of the water column by employing 70 kHz (ES70-18CD) and 333 kHz (ES333-7CDK) center frequency transducers. These are not optimal for the detection and quantification of fish, due to the acoustic frequencies and pulse repetition frequencies employed. However, to expand glider capabilities and perform fish acoustic assessments in the future, a 200 kHz transducer can be adapted. Another potential approach is operating the echo sounder in CW mode (narrowband) and utilizing transducers with a wider beamwidth.

The results presented here corroborate the effectiveness of gliders equipped with passive acoustic monitoring devices in collecting long-term marine mammal data and anthropogenic noise across a wide geographical area, with minimal disturbance to animals [11,37]. The novel Seaglider^®^-integrated hydrophone used in this study was able to record cetacean vocalizations. The production of time series datasets during periods of unknown animal distribution contributes to the growing body of knowledge on marine mammal distribution in the Norwegian sea outside traditional summer survey periods. Although it is generally known that sperm whales are often found along the continental slope, this fact had not yet been documented in the Norwegian Sea and outside summer months. Furthermore, recordings of humpback whale occurrence beyond the months previously reported in the literature for low-latitude migration shows that not only can gliders equipped with passive acoustic equipment document species distribution, but they can also address gaps in areas and periods of the year for which there is a severe lack of information [44,73]. Advantages of using gliders for recording cetaceans include cost efficiency, the capability of working under adverse weather conditions, and the collection of passive acoustic data over periods of weeks to months (e.g., [11]). Gliders are quiet monitoring platforms with a much lower acoustic impact on the ecosystem compared to traditional ship-based survey systems. As for zooplankton data collection, this is not achievable with traditional research platforms such as research vessels, but it is essential to detect ecosystem changes brought about by anthropogenic or natural disturbances (i.e., storms).

Although both the Seaglider^®^ and Wave Glider were equipped with hydrophones, only the data recorded by the Seaglider^®^ were processed due to the large amount of data collected by each platform and time/budget constraints associated with data processing. Furthermore, considerations would have to be made for sampling that occurred at a single depth (Wave Glider) and sampling across multiple depths of the water column (Seaglider^®^), which would likely not be comparable due to the latter recording data at a different spatial plane. This study also showed the potential of gliders to detect and record anthropogenic noise, and when overlapping with cetacean vocal activity, how noise may represent a potential stressor for marine mammals. Underwater noise radiated by ships was previously recorded in LoVe and is one of the main sources of anthropogenic marine noise [74,75]. On the technical side, the placement of acoustic sensors onboard autonomous vehicles will influence the quality of the data obtained. In the first Wave Glider deployment, the hydrophone was installed on the keel of the surface float, resulting in the sound of the wave breaking on the keel saturating the spectrogram for the duration of the survey. Yet, we observed that installing the hydrophone on a towed cable attached to the subsurface glider decreased drastically the recording of the self-noise produced by the float. 

Regarding ocean modeling, our results showed the importance and complementary role of gliders in increasing the accuracy of the ocean circulation models. We noted that by assimilating a few sporadic glider observations, our model’s performance was greatly improved. A systematic glider-based observing system can be an important complement to better constrain small-scale circulation features [53]. Additionally, our experiments demonstrated that high-resolution observations combined with an eddy-resolving multi-scale model could improve simulation skills towards capturing multiscale flow dynamics, which is fundamental in the understanding of mass and energy transport mechanisms in the oceanic environment. The GLIDER project demonstrated that a suite of autonomous vehicles can collect physical oceanographic parameters alongside biological data across multiple ecosystem components in the coastal Arctic. The productive waters of the LoVe region are shared by ecological important low (phytoplankton and zooplankton) and high (marine mammals) trophic groups and an increasing number of human stakeholders. High spatial and temporal resolution data collected with a small environmental footprint and made available by the GLIDER data management platform make it possible to capture ecosystem dynamics, important towards the realization of an ecosystem-based management approach in the region and elsewhere. The integration between the current underwater observatory system in LoVe and gliders should be prioritized to form an ecosystem observatory module network able to provide unique marine research and monitoring capabilities.

## Figures and Tables

**Table 1:** Sensors integrated on the gliders for the 2018 LoVe deployment.

Manufacturer	Sensor	Variables
Wave Glider		
Kongsberg Maritime	WBT Mini echo sounder (ES70-18CD, ES333-7CDK split	Acoustic backscatter
LiCor	Light Sensor Li1500	Photosynthetically active radiation in air
SAIV	SAIV-SD204	Salinity, temperature, density
Seapoint (on SAIV CTD)	Seapoint Turbidity Meter	Turbidity
Seapoint (on SAIV CTD)	Seapoint Chlorophyll Fluorometer	CHl-*a*
Aanderaa Xylem	Optode 4831	Oxygen
Aanderaa Xylem	Submersible CO_2_ 4797	*p*CO_2_
Airmar	Airmar 200WX	Wind speed and direction, air temperature, barometric pressure
Ocean Instruments	Soundtrap HF hydrophone (parts of the deployment)	Underwater sound
Sailbuoy		
Kongsberg Maritime	WBT Mini echo sounder (ES333-7CDK split)	Acoustic backscatter
Neil Brown Ocean Sensors	NBOSI Cabled CT sensor	Salinity, temperature, density
Aanderaa Xylem	Optode 4831	Oxygen, temperature
Seaglider^®^		
Sea-Bird Scientific	SeaBird CT-Sail	Temperature, conductivity
Discontinued (formerly Kongsberg Maritime Contros)	CONTROS HydroFlash^®^ O_2_ optode	Oxygen
Sea-Bird Scientific	SeaOWL UV-A	Total particulate concentration
Sea-Bird Scientific	SeaOWL UV-A	CHl-*a*
Sea-Bird Scientific	SeaOWL UV-A	FDOM fluorescence
JASCO	Observer/AMARG4	Underwater sound

**Table 2:** Average and standard deviation of monthly temperature, salinity, and oxygen saturation measured from the Sailbuoy glider, from April to July 2018.

Month	Temperature (°C)	Salinity (ppt)	O_2_ Saturation (%)
April	4.5 +/− 0.7	33.6 +/− 0.2	101.2 +/− 2.1
May	8.6 +/− 0.4	33.5 +/− 0.0	110.0 +/− 1.6
July	14.1 +/− 0.1	34.5 +/− 0.0	104.7 +/− 0.6
